# Commissioning and quality assurance for intensity‐modulated radiotherapy with dynamic multileaf collimator: Experience of the Pontificia Universidad Católica de Chile

**DOI:** 10.1120/jacmp.v5i3.1982

**Published:** 2004-10-21

**Authors:** Carlos Daniel Venencia, Pelayo Besa

**Affiliations:** ^1^ Centro de Cáncer “Nuestra Señora de la Esperanza” Pontificia Universidad Católica de Chile Diagonal Paraguay 319 Santiago Chile

**Keywords:** intensity‐modulated radiotherapy, commissioning, quality assurance, implementation, IMRT protocol

## Abstract

The objective of this paper is to present our experience in the commissioning and quality assurance (QA) for intensity‐modulated radiotherapy (IMRT) using the dynamic multileaf collimator (dMLC), sliding window technique. The connectivity and operation between all IMRT chain components were checked on the Varian equipment. Then the following tests were performed: stability of leaf positioning and leaf speed, sensitivity to treatment interruptions (acceleration and deceleration), evaluation of standard field patterns, stability of dMLC output, segmental dose accuracy check, average leaf transmission, dosimetric leaf separation, effects of lateral disequilibrium between adjacent leaves in dose profiles, and multiple carriage field verification. Standard patterns were generated for verification: uniform field, pyramid, hole, wedge, peaks, and chair. Weekly QA protocol includes sweeping gap output, garden fence test (narrow bands, 2 mm wide, of exposure spaced at 2‐cm intervals), and segmental dose accuracy check. Monthly QA includes sweeping gap output at multiple gantry and collimator angles, sweeping gap off‐axis output, picket fence test (eight consecutive movements of a 5‐cm wide rectangular field spaced at 5‐cm intervals), stability of leaf speed and leaf motor current test (PWM test). Our patient QA procedure consists of an absolute dose measurement for all treatment fields in the treatment condition, analysis of actual leaf position versus planned leaf position (dynalog files) for each treatment field, film relative dose determination for each field, film relative dose determination for the plan (all treatment fields) in two axial planes, and patient positioning verification with orthogonal films. The tests performed showed acceptable results. After over one year of IMRT treatment, the routine QA machine checks confirmed the precision and stability of the IMRT system.

PACS number: 87.53.Xd, 87.53.Dq, 87.53.Mr

## I. INTRODUCTION

Intensity‐modulated radiotherapy (IMRT) using inverse treatment planning can improve dose distributions compared with conformal radiotherapy planning (3D‐CRT).^(1)^ Multileaf collimator (MLC)–based IMRT can be delivered with two main modalities: segmental IMRT (step and shoot) and dynamic IMRT (sliding window). For the step‐and‐shoot modality, the MLC shape remains constant while the beam is on and changes while the beam is off. For the sliding window, each leaf pair moves continuously, unidirectionally, and with independent speed while the beam is on.

The clinical implementation of IMRT requires special commissioning, including machine and patient‐related routine quality assurance (QA) beyond the ones currently performed for 3D‐CRT with the MLC.^(^ ^2^ ^–^ ^7^ ^)^ Many components of this new technology, both hardware and software, are recent and their potential errors are unknown. There are no international guidelines for IMRT commissioning and QA. Recent publications describe some new procedures required to carry out the implementation of IMRT^(8)^; however, there are few reports that describe special procedures or provide guidance in introducing IMRT.^(^ ^9^ ^,^ ^10^ ^)^ Radiotherapy departments wishing to implement IMRT need a commissioning and QA protocol.

The purpose of this paper is to present our experience with the implementation of IMRT commissioning and QA with dynamic multileaf collimator (dMLC) using the sliding‐window technique.

## II. METHODS AND MATERIALS

### A. The IMRT system

All the following equipment was from Varian Medical Systems, Inc., Palo Alto, CA. A linear accelerator Clinac 21EX, equipped with a 120‐leaf MLC Millennium, was used for dynamic IMRT. The treatment energy used was 6 MeV photons. Virtual simulation was achieved with SomaVision. The 3D treatment‐planning system (TPS) was Cadplan, and the inverse‐planning system was Helios. All these systems were interfaced with Varis.

Relative dosimetry was achieved by using RIT113 software (Radiological Imaging Technology, Inc., Colorado Springs, CO), with Kodak EDR2 and X‐Omat V Ready‐Pack film (Eastman Kodak Company, Rochester, NY) and film scanner VIDAR VXR‐12 (VIDAR Systems Corporation, Herndon, VA). The optical density versus absorbed dose to water curve was obtained for both types of films. Film was irradiated perpendicular to the incident beam (perpendicular configuration) in a solid water phantom at 5 cm depth, with only one exposure per film. To reduce the low energy scatter to the film, 0.5‐mm lead sheets were placed at 1.2 cm on both sides.^(11)^ Film dosimetry was used only for relative measurements. An automatic film processor (FPM 2800, FUJI Photo Film Co., Tokyo, Japan) was used to develop the film. To reduce the variability of working conditions, both the calibration and commissioning dosimetry measurements were performed within a single irradiation and developing session.

For absolute dosimetry a 0.6 cm^3^ farmer‐type ionization chamber (PTW‐FREIBURG W30001, Freiburg, Germany) was used. The phantoms used were solid water slabs (Gammex Inc., Middleton, WI) and the Benchmark IMRT Phantom (MED‐TEC Inc., Orange City, IA).

### B. Commissioning

After standard accelerator commissioning we proceeded to verify the connectivity and operation between all the components of the IMRT chain. The dosimetric precision of IMRT with dMLC is critically dependent on the accurate positioning and reproducibility of the gap between opposing leaves.^(5)^ The initial adjustment of the MLC is crucial because it establishes the baseline for future comparisons.^(12)^ During the initial commissioning process, the mechanical and dosimetric control tests listed in Table 1 were performed.

**Table 1:** Mechanical and dosimetric tests done during commissioning and when the machine undergoes major maintenance

1. Mechanical Checks
1.1 Stability of the dMLC
1.2 Stability of leaf speed
1.3 Sensitivity to treatment interruptions (acceleration and deceleration)
1.4 Evaluation of standard patterns, Varian test^(13)^
• synchronized segmented stripes
• nonsynchronized segmented stripes
• x wedges
• Y wedges
• pyramids
• complex field A
• complex field B
1.5 Evaluation of fields with multiple carriage position
2. Dosimetric Checks
2.1 Stability of dMLC output sweeping gap test (central axis (CAX) vs. gantry and collimator angles)
2.2 Segmental dose accuracy check
2.3 Determination of average leaf transmission
2.4 Determination of dosimetric leaf separation
2.5 Effects of lateral disequilibrium in dose profile between adjacent leaves

#### B.1 Mechanical checks

##### B.1.1 Stability of the dMLC

**Figure 1:**
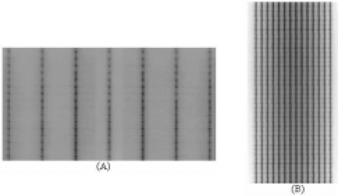
(a) The picket fence and (b) garden fence tests are used to check the stability of the dMLC mode and the reproducibility of the gap between the leaves.

To check the stability of the dMLC mode and the reproducibility of the gap between leaves, two tests were carried out: the picket fence test (Fig. 1(a) and the garden fence test (Fig. 1(b), so called because the diagram obtained on the exposed film resembles a fence. The picket fence test consists of eight consecutive leaf movements of a 5‐cm wide rectangular field spaced at 5‐cm intervals; the field information is contained in three separate test files which are run in sequence.^(13)^ This test field was exposed on a Ready‐Pack film (33 cm×43 cm) at 100 cm source‐to‐surface distance (SSD), placed over the treatment couch with 2 cm solid water buildup. The garden fence test consists of a narrow band (2 mm wide) spaced at 2‐cm intervals.^(5)^

The garden fence test was performed at four gantry angle positions (0°, 90°, 180°, and 270°). This test field was exposed on Ready‐Pack film (25.4 cm×30.5 cm) fixed to the collimator tray holder. Each leaf match line was analyzed either visually or using full‐width half‐maximum.

##### B.1.2 Stability of leaf speed

The stability of leaf speed was verified using a test field that requires the leaf pair to move at seven constant speeds, generating a stepwise homogeneous dose delivery of well‐defined intensity.^(4)^ This file was generated using Shaper software (Varian Medical Systems, Inc., Palo Alto, CA). The stability of the different speed levels was analyzed, comparing the uniformity of each profile to the open field profile.

##### B.1.3 Sensitivity to treatment interruptions

During the leaf speed test, two beam interruptions were introduced to test the potential effect of acceleration and deceleration.^(4)^ The stability of the intensity levels was analyzed, comparing the uniformity of each profile to the open field.

##### B.1.4 Evaluation of standard patterns

Quality assurance test patterns provided by Varian^(13)^ were performed. The tests are designed to achieve a qualitative analysis of the position accuracy of the leaf, kinetic properties of the dMLC, and a dosimetric evaluation of fractional dose delivery.

##### B.1.5 Evaluation of fields with multiple carriage positions

The leaves of the MLC are mounted on a carriage. The maximum leaf motion relative to the corresponding carriage is 15 cm. Because the carriage remains fixed during irradiation, fields larger than 15 cm are delivered with two or three overlapping subfields with different carriage positions. Large fields are set to evaluate accuracy of multiple carriage delivery.

#### B.2 Dosimetric checks

##### B.2.1 Output stability with dMLC

The output stability with the dMLC was verified with a sweeping gap of 1.0 cm creating a uniform field of $20\times 20\;{\text{cm}}^{2}$. The ion chamber was placed at the central axis (CAX) and off‐axis, at positions ±5 cm; the measurement was normalized to the reference dose. To evaluate the possible effect of gravity, four different gantry angles were used (0°, 90°, 180°, and 270°). To determine whether the leaf gap changed between runs, measurements were taken using various gap widths.

##### B.2.2 Segmental dose accuracy check

Segmental dose checks allow for proving that interruptions sent by the control system are carried out correctly. In this test, a multiple beam segment field was used, and the dose of the first segment was measured and normalized to the reference dose. The first segment consisted of a $10\times 10\;{\text{cm}}^{2}$ MLC field.

##### B.2.3 Determination of average leaf transmission

Average leaf transmission was determined with the ionization chamber and radiographic film as the ratio of the dose delivered through a fully closed and fully opened static MLC field. The ion chamber determination was obtained in a solid water phantom at the depth of maximum dose, averaging interleaf and midleaf transmission. The determination of leaf transmission with radiographic film was obtained in a solid water phantom, with films placed at 5‐cm depth, perpendicular to the incident beam, averaging the dose profile perpendicular to the leaf direction in the center of the field. The transmission of all the leaves was verified following the factory‐recommended procedure.

##### B.2.4 Determination of dosimetric leaf separation

Dosimetric leaf separation was determined using films placed at 100 cm from the source, at 5‐cm depth in a solid water phantom.^(5)^ Multiple static MLC fields with gap widths from 0 cm to 10 cm were used. All films were scanned and dose profiles in the direction of leaf movement were obtained. The dosimetric leaf separation was determined by extrapolating the average integral profile dose versus the MLC gap width to the zero dose.

##### B.2.5 Effects of lateral disequilibrium

The effect of lateral disequilibrium between adjacent leaves was measured using the leaf speed stability test, analyzing the profile perpendicular to the direction of the leaf motion.^(4)^

#### B.3 Dosimetric verification for the total system

**Figure 2:**
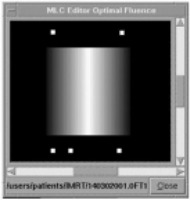
Registration points on a pyramid optimal fluence. Registration points are created using in‐house software, which reads the optimal fluence for the treatment field and generates the registration points outside of the treatment area.

To determine the accuracy of the dose distribution with dMLC for the combination of the TPS and LINAC, computer‐generated calculations were compared with film‐measured dose distributions. Standard patterns were created manually for comparison by editing the optimal fluence file with a text editor, and assigning intensity values to the corresponding fluence matrix components (cell index value). The fluence patterns created for verification were uniform field, pyramid, hole, wedge, peaks,^(3)^ and chair.^(2)^ To match calculated to film‐measured isodoses, registration points were created using in‐house software. This software reads the optimum fluence of the treatment field and generates the registration points (Fig. 2). The registration points are outside of the irradiated area and do not interfere with the measurements.

Dose distribution was calculated by the TPS on a coronal plane in a flat phantom at 5‐cm depth using a 2.5‐mm calculation grid. Comparisons between calculated and measured doses for different standard patterns were performed by dose profile and superposition of isodoses.

### C. Machine quality control

To guarantee the ongoing stability and geometrical precision of the dMLC, it is necessary to add special tests to the standard QA program.^(^ ^2^ ^,^ ^4^ ^,^ ^6^ ^)^ The tests and frequency required for each one are shown in Table 2. In addition, the weekly and monthly checks must be carried out each time preventive maintenance for the MLC is performed.

**Table 2:** Protocol for QA of the dMLC and the frequency with each test is performed

1. Weekly
1.1 Sweeping gap output (gantry and collimator angle 0°)
1.2 Garden fence test (4 h post‐initialization of the MLC)
1.3 Segmental dose accuracy check
2. Monthly
2.1 Sweeping gap output versus gantry angle (0°, 90°, 180° and 270°, collimator angle 0°)
2.2 Sweeping gap output off‐axis (±5 cm)
2.3 Picket fence test
2.4 Stability of leaf speed
2.5 Leaf motor current test (pulse‐width modulator (PWM) test from Varian)

The stability of leaf speed and leaf motor current test (PWM test) provided by the manufacturer directly determines the amount of current needed to move a leaf a certain distance, in a certain time, at different positions.

### D. Patient treatment verification

**Figure 3:**
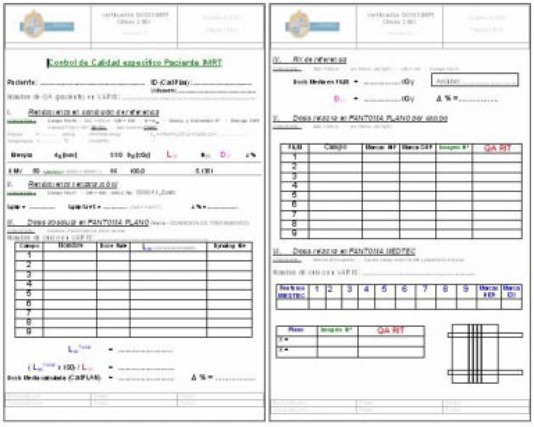
The IMRT form must be completed for each patient as part of the pretreatment QA program.

Pretreatment verification for IMRT is performed before the patient starts therapy. All verifications performed are shown in Table 3. A checklist, IMRT QA form, must be completed for each patient (Fig. 3).

**Table 3:** Protocol for patient pretreatment verification

1. Absolute dose for all treatments fields (treatment condition)
2. Analysis of the dynalog files for each treatment field
3. Relative dose for each treatment field
4. Relative dose for all treatment fields in two axial planes
5. Treatment position verification (two orthogonal films)

#### D.1 Absolute dose for all treatment fields

The IMRT fields are imported from the patient's plan into the phantom used for measurements. Dose distributions are calculated and mean dose for a volume, which represents the ion chamber, is obtained from the dose volume histogram (DVH). The calculated dose is compared with the measured dose. A 0.6 cm^3^ farmer‐type ionization chamber is used for the measurement. An acceptance criterion for the absolute dose was developed; the absolute dose is considered adequate if the difference between the calculated and the measured dose is <3%. If the difference between measured and calculated dose is 3% to 5%, the plan has to be verified; if >5%, the plan is rejected.

#### D.2 Analysis of the dynalog files

The dynamic log files, created by the MLC controller at the end of every IMRT field delivery, are analyzed using Dynalog File Viewer software (Varian Medical Systems, Inc., Palo Alto, CA), which is part of the MLC Varian workstation software. This file contains information about the planned versus actual position for all leaves every 50 ms while the beam is on. The software generates data tables and plots an error histogram (which shows information for all leaf position deviations), error RMS (which shows the calculated root‐mean‐square value for individual leaf deviations), and beam hold‐off versus time plot (which shows beam hold‐off during dynamic treatment). For each field the error histogram result is considered acceptable if 95% or more of the error counts (number of leaf position deviations) have misplacements <0.1 cm and there is no error count with misplacements >0.3 cm. The largest accepted RMS is 0.05 cm. The maximum number of beam hold‐offs accepted per field is 2. Error histogram and error RMS are saved for each IMRT field. The data obtained are also used to screen for initial MLC motor failure and determine the need for preventive replacement.

#### D.3 Relative dose for each treatment field

**Figure 4:**
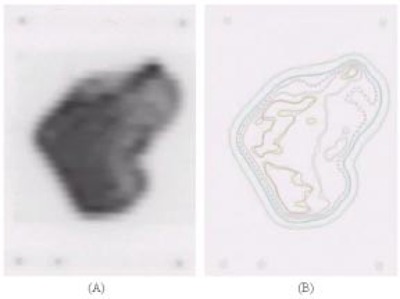
(a) Film from an IMRT treatment field and (b) isodose superposition from calculated and measured isodoses.

To obtain the relative dose for each treatment field, the fluence for each field is imported into a flat phantom with normal incidence. Dose distribution is calculated on a coronal plane at 5 cm depth with Cadplan. Registration points outside of the treatment field are used to match measured and calculated doses. Comparisons between calculated and measured isodoses are carried out by overlapping and/or subtracting curves (Fig. 4).

#### D.4 Relative dose for all treatment fields

**Figure 5:**
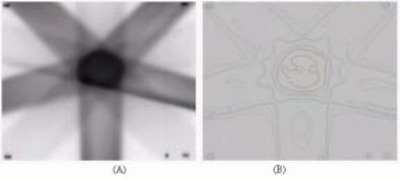
(a) Film from an IMRT treatment plan and (b) multiple fields and isodose superposition from calculated and measured isodose.

The total plan is imported into a benchmark IMRT phantom. Two additional fields are added with the registration points for position information. Relative doses are calculated on two axial planes. Comparisons between calculated and measured film isodoses are carried out by overlapping and/or subtracting curves (Fig. 5).

## III. RESULTS AND DISCUSSION

**Figure 6:**
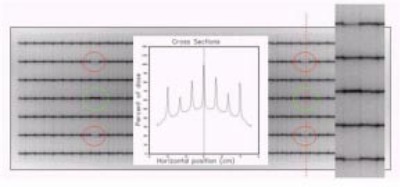
Garden fence test with errors. To evaluate the sensitivity to detect leaf pair errors, several small positional errors were introduced in the sequence of the garden fence test. Errors in individual leaf position or gaps as small as 0.05 cm can easily be detected.

The initial adjustment of the MLC is crucial in producing the reference values that will allow the detection of future MLC errors. Picket fence and garden fence tests were designed to accurately detect leaf position errors. To evaluate the sensitivity of detecting leaf pair errors, several errors were introduced into the garden fence sequence^(4)^ by editing the leaf motion sequence. Errors in individual leaf positions or gaps as small as 0.05 cm could easily be detected (Fig. 6). The garden fence, picket fence, and the PWM tests allowed for the detection of problems with the leaf components (motor, screw, plastic nut, or spring).

The profiles obtained from the speed stability test for all intensity levels were uniform; dose differences to the open field profile were always below 2%. When unscheduled interruptions were introduced during treatment delivery, the dose profiles with these interruptions were not different from the tests without interruptions. Leaf speed remained stable, and dose delivered was not affected by the interruptions.

A qualitative analysis of all standard Varian pattern tests shows straight field boundary and match lines between different intensity segments. For all cases, match line segments fell within the positional limit error of ±1.0 mm established by the manufacturer.

**Figure 7:**
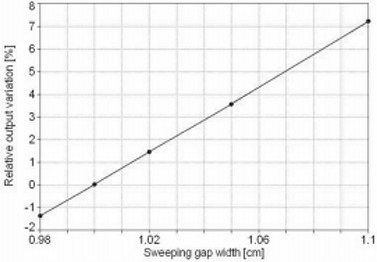
Sweeping gap relative dose versus gap width. Output variation for sweeping gap versus the gap width. A change of 1.5% in the output corresponds to a 0.2‐mm gap width variation.

The variation in the output of the sweeping gap versus the gap width is shown in Fig. 7. A variation of 1.5% in the output corresponds to a 0.2‐mm error in the gap width. After more than a year, the 1‐cm sweeping gap output test had a maximum deviation of less than 1.5%, including at different gantry angles.

Segmental dose measurements show variations of less than 0.5% after more than a year.

**Figure 8:**
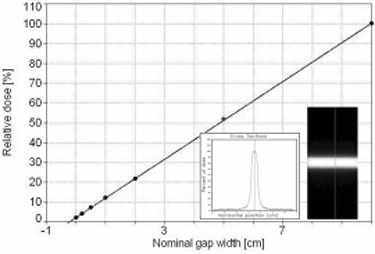
Dosimetric leaf separation was determined with film measurement, extrapolating to zero dose the average integral profile dose versus the MLC gap width.

The average leaf transmission found for 6 MeV was 1.4% with ion chamber and 1.6% with film; a value of 1.5% was adopted. The dosimetric leaf separation determined by film for 6 MeV was 2.0 mm (Fig. 8).

A summary of the parameters used for the Cadplan configurations is listed in Table 4.

**Table 4:** Parameters for Cadplan v6.2.7, IMRT with 6 MeV

Leaf tolerance	0.15 cm
Dose rate for dMLC	300 MU/min
Dosimetric leaf separation in dMLC	0.20 cm
Minimum leaf gap in dMLC	0.06 cm
Average transmission	1.5%

**Figure 9:**
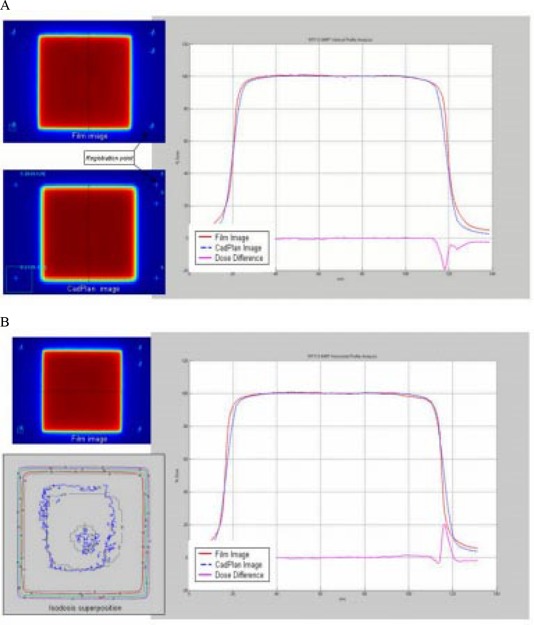
(a) Comparison between TPS calculated and measured relative dose for the uniform test field in the direction of leaf movement. Profile superposition shows an agreement better than 1% in the HD‐LG region. (b) Profile superposition in a perpendicular direction to leaf movement shows an agreement better than 1% in the HD‐LG region.

Comparison between TPS calculation and measured relative dose was done for all standard patterns. For the uniform test field (Fig. 9), profile superposition shows an agreement better than 1%, in the high dose‐low gradient (HD‐LG) region, both in the direction of leaf movement and in the perpendicular direction. The localization of the registration points used for the superposition of the images is shown in Fig. 9. Dose differences in the high‐gradient region increase due to different film resolutions and the calculation grid size.

**Figure 10:**
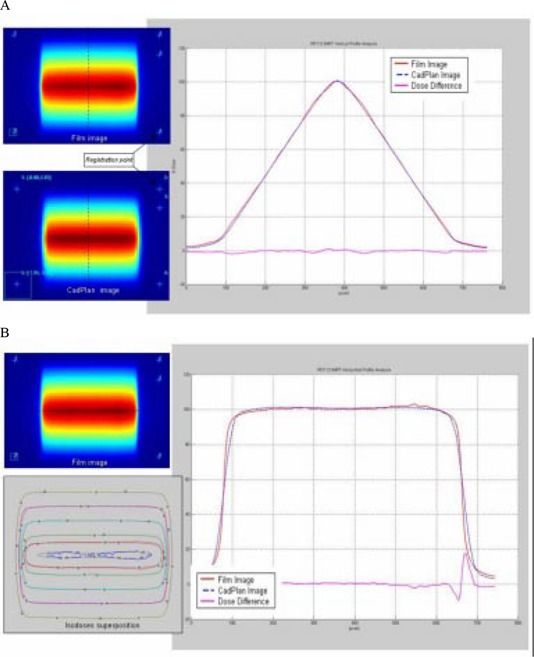
(a) Comparison between TPS calculated and measured relative dose for the pyramid test field in the direction of leaf movement. Profile superposition shows an agreement better than 1% in the HD‐LG region. (b) Comparison in a perpendicular direction to leaf movement shows dose difference similar to the uniform test field. Isodose superposition shows agreement better than 4% dose difference and 3‐mm distance to agreement.

For the pyramid test field (Fig. 10), the profile superposition shows an agreement better than 1% in the direction of leaf movement; in the perpendicular direction the dose difference is similar to the uniform test field. The isodose superposition for the pyramid field shows good agreement, better than 4% dose difference and 3‐mm distance to agreement.

**Figure 11:**
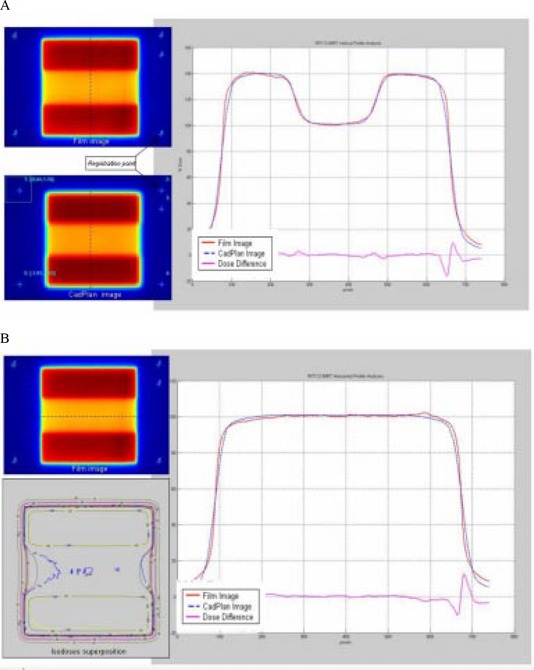
(a) Comparison between TPS calculated and measured relative dose for the hole test field in the direction of leaf movement. Profile superposition shows an agreement better than 3% in the HD‐LG region. (b) Comparison in a direction perpendicular to leaf movement shows a dose difference similar to the uniform test field. Isodose superposition shows agreement better than 4% dose difference and 3‐mm distance to agreement.

The hole test field (Fig. 11) shows two different dose levels; the profile superposition in the direction of leaf movement shows an agreement better than 3% in the HD‐LG region. In the perpendicular direction the dose difference is similar to the uniform test field. Isodose superposition for the hole test field shows good agreement, better than 4% dose difference and 3‐mm distance to agreement.

**Figure 12:**
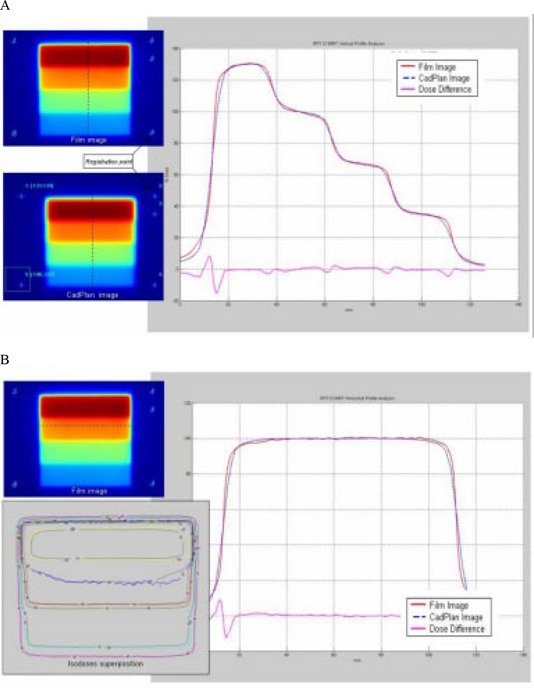
(a) Comparison between TPS calculated and measured relative dose for the wedge test field in the direction of leaf movement. Profile superposition shows an agreement better than 2% in the HD‐LG region. Dose difference increases at the edge of each dose level. (b) Comparison in a direction perpendicular to leaf movement shows dose difference similar to the uniform test field. Isodose superposition shows agreement better than 4% dose difference and 3‐mm distance to agreement.

For the wedge test field (Fig. 12), profile superposition in the direction of leaf movement shows an agreement better than 2% in the HD‐LG region for the four dose levels. Dose differences increase at the edge of each dose level.

**Figure 13:**
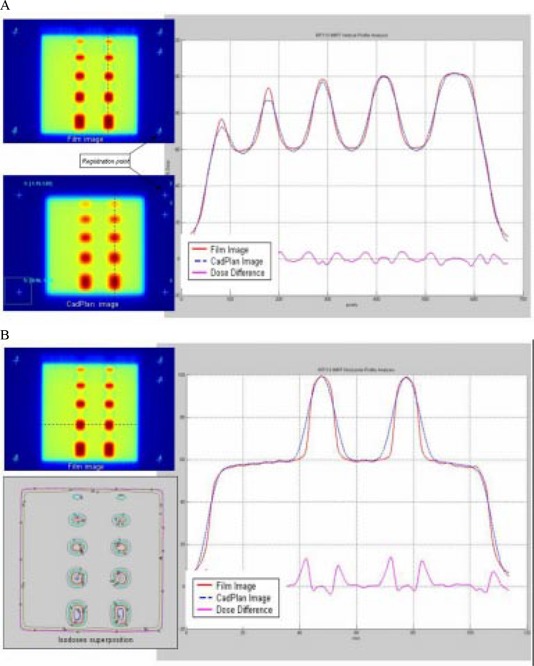
(a) Comparison between TPS calculated and measured relative dose for the peak test field in the direction of leaf movement. Profile superposition shows an agreement better than 2% for 15‐mm and 10‐mm peak width, 3% for 7.5‐mm peak width, 5% for 5‐mm peak width, and 7% for 2.5‐mm peak width. (b) Comparison in a direction perpendicular to leaf movement; profile superposition shows an agreement better than 2%. Dose difference increase in the high gradient region.

The peak test field (Fig. 13) shows peaks of different widths: 2.5 mm, 5 mm, 7.5 mm, 10 mm, and 15 mm in the direction of leaf movement with 10‐mm height. Dose normalization was done at the center of the 10‐mm width peak. Profile superposition shows an agreement better than 2% for 15‐mm and 10‐mm width peaks, 3% for 7.5‐mm width peak, 5% for 5‐mm width peak, and 7% for 2.5‐mm width peak. Dose profiles in the perpendicular direction show an agreement better than 2% in the HD‐LG and the dose difference increases in the HG region.

**Figure 14:**
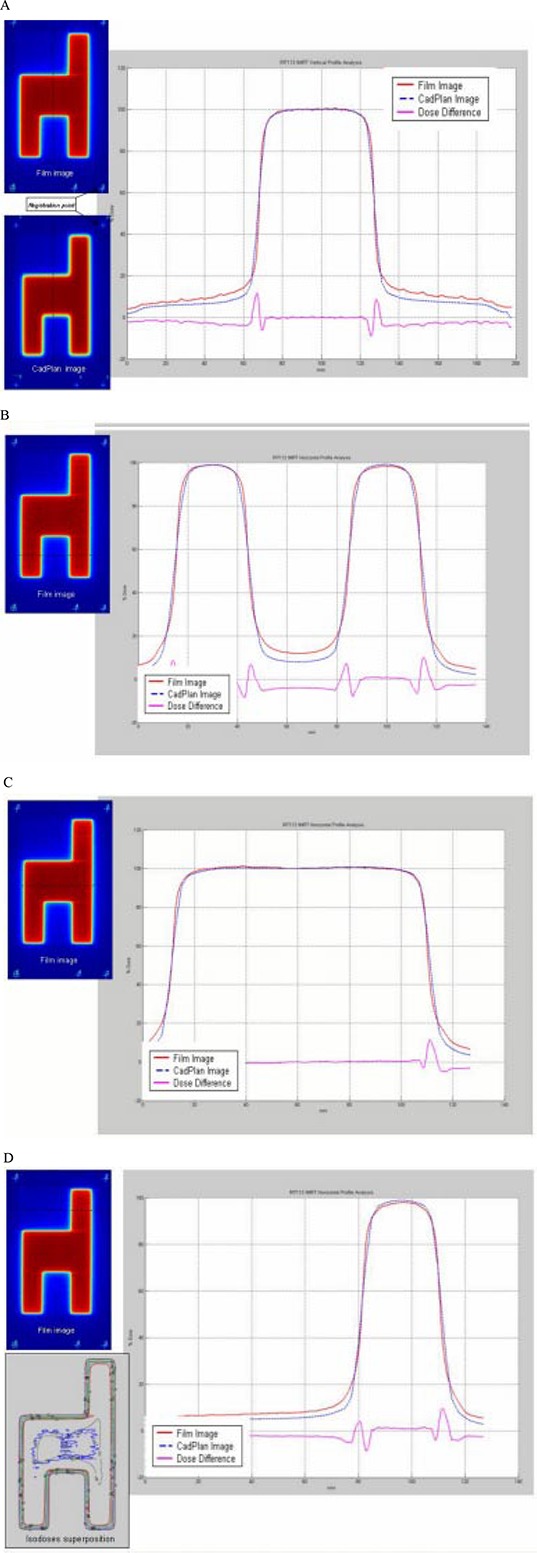
(a) Comparison between TPS calculated and measured relative dose for the chair test field in a direction perpendicular to leaf movements. Differences in the low‐dose region increase up to 5%. (b), (c), (d) Profile comparisons in the direction of leaf movement. Dose differences increase up to 5%.

For the chair test field (Fig. 14) the dose profile superposition in the direction of leaf movement and the perpendicular direction show agreements similar to the uniform test field. In the low‐dose region differences increase up to 5%, probably due to the film's sensitivity to scatter radiation.

The dose rate is adjusted depending on the number of beam hold‐offs for a dynamic field. If more than two beam hold‐offs are detected for a given treatment field, the dose rate is reduced by 100 MU/min.

**Figure 15:**
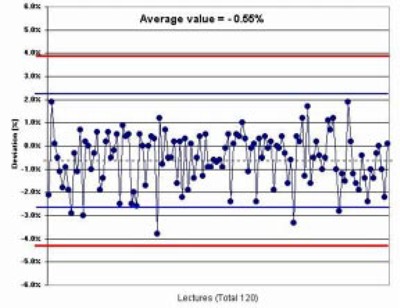
Measured versus calculated dose in IMRT patients. Comparison between the calculated and ion chamber measured absolute dose for 120 IMRT patients.

The variations of the calculated absolute dose for all treatment fields with the ion chamber measurement, for the first 120 patients, were within the acceptable criterion (Fig. 15). The average variation value was −0.55%.

## IV. CONCLUSION

All mechanical and dosimetric tests have proved very useful and detected only minor deviations. After more than one year of routine use of our QA protocol for dMLC IMRT, we have not detected any deviation in the MLC position. The implementation of IMRT must not be underestimated or simplified. Treatment‐planning system configuration parameters must be measured. The TPS calculated dose and the measured absolute dose should not deviate more than 3% to ensure safe treatment. It is necessary to maintain strict criteria to compare measured and calculated values. The patient pretreatment QA requires significant machine time for measurements and must be completed before treatment starts.

In the future, in order to reduce patient pretreatment QA time, an independent monitor unit calculation program^(^ ^14^ ^,^ ^15^ ^)^ can be evaluated. Also, introduction of new analyzing tools, such as DTA (distance to agreement)^(16)^ or γ (gamma factor),^(17)^ can be useful to better quantify the comparison between measured versus calculated dose distributions.

Every institution should adopt a QA protocol. The QA protocol presented here has proved adequate, and with it we have had no patient complications attributed to IMRT delivery.

## ACKNOWLEDGMENTS

The authors wish to thank Chen‐Shou Chui, PhD, Doracy Fontenla, PhD, and Yakov Pipman, PhD, for reviewing this paper.
